# Supplementary material for: Structural basis for virulence regulation in Vibrio cholerae by unsaturated fatty acid components of bile
Source: Commun Biol. 2019 Nov 28;2:440. doi: 10.1038/s42003-019-0686-x (PMC6882843; doi:10.1038/s42003-019-0686-x)
Supplement: Supplementary file 1 — Supplementary Information [file 42003_2019_686_MOESM1_ESM.pdf]

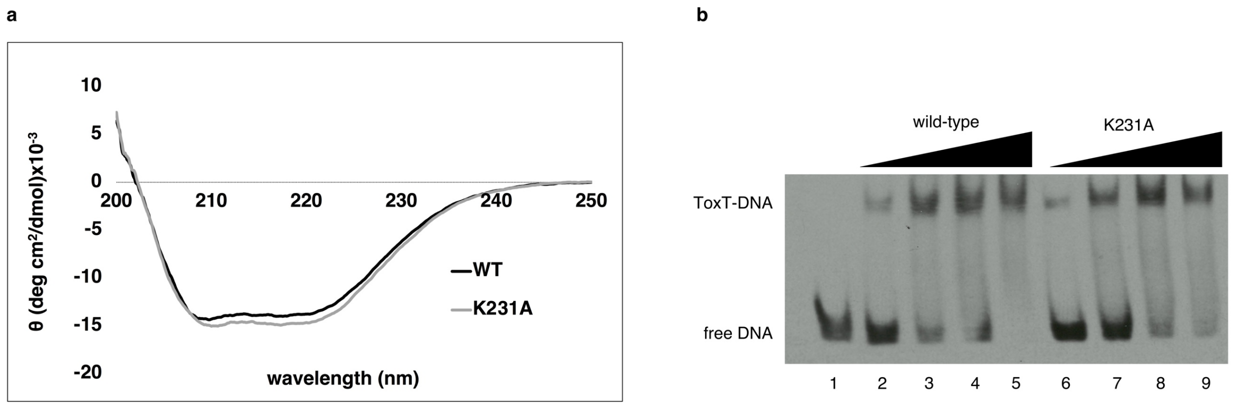


**Supplementary Figure 1.** K231A $ToxT_{ENV256}$ has the same secondary structure and binds DNA as well as wild-type. **a**, Circular dichroism spectra of purified wild-type (black) and K231A (gray) $ToxT_{ENV256}$. **b**, Electrophoretic mobility shift assay showing the specific binding of wild-type and K231A $ToxT_{ENV256}$ to a DIG-labeled 84 bp segment of dsDNA containing the ToxT-binding sites from the *tcpA* promoter. All lanes contain 9 nM of DIG-labeled DNA. Lane 1, free DNA; lane 2, 0.098 μM wild-type $ToxT_{ENV256}$; lane 3, 0.195 μM wild-type $ToxT_{ENV256}$; lane 4, 0.39 μM wild-type $ToxT_{ENV256}$; lane 5, 0.78 μM wild-type $ToxT_{ENV256}$; lane 6, 0.098 μM K231A $ToxT_{ENV256}$; 7, 0.195 μM K231A $ToxT_{ENV256}$; lane 8, 0.39 μM K231A $ToxT_{ENV256}$; lane 9, 0.78 μM K231A $ToxT_{ENV256}$.

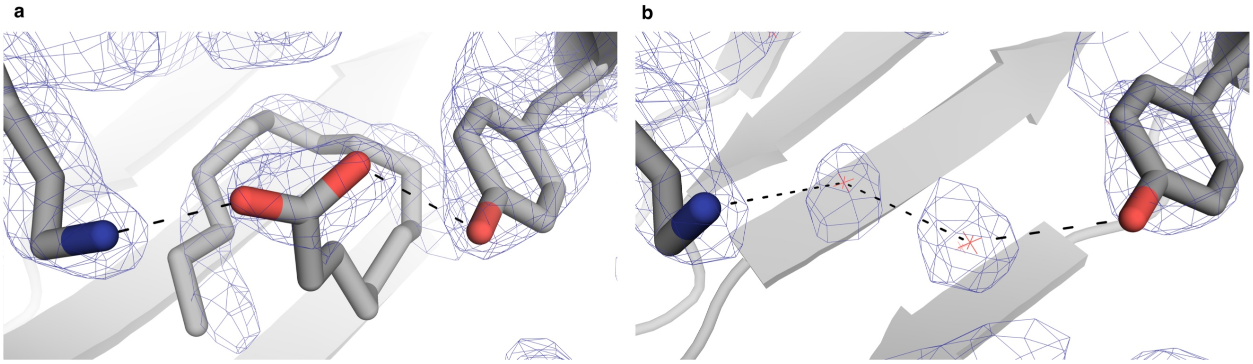


**Supplementary Figure2.** Electron density in the UFA-binding pocket of UFA-bound and apo $ToxT_{ENV256}$ K231A. **a**, 2FO-FC electron density map of the UFA-binding pocket in the UFA-bound $ToxT_{ENV256}$ structure. Electron density is contoured to 1.5 $\sigma$. **b**, 2FO-FC electron density map of the UFA-binding pocket in the apo $ToxT_{ENV256}$ structure. Electron density is contoured to 1.5 $\sigma$.

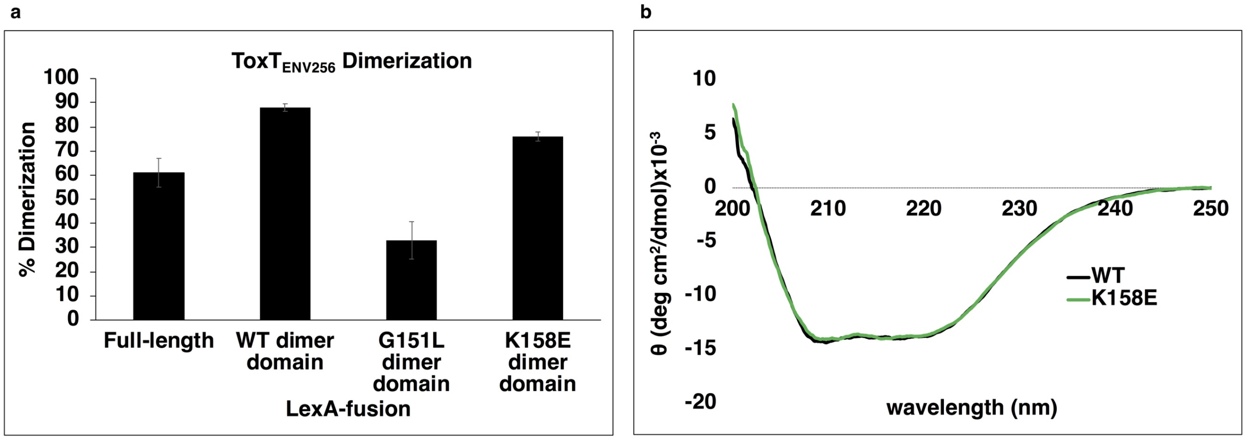


**Supplementary Figure 3.** *In* vivo Dimerization of the $ToxT_{ENV256}$ regulatory domain and secondary structure of purified K158E $ToxT_{ENV256}$. **a**, LexA-fusion bacterial two-hybrid dimerization assay of $ToxT_{ENV256}$ dimer interface mutants. Error bars are of the standard deviation. **b**, Circular dichroism spectra of purified wild-type (black) and K158E (green) $ToxT_{ENV256}$.

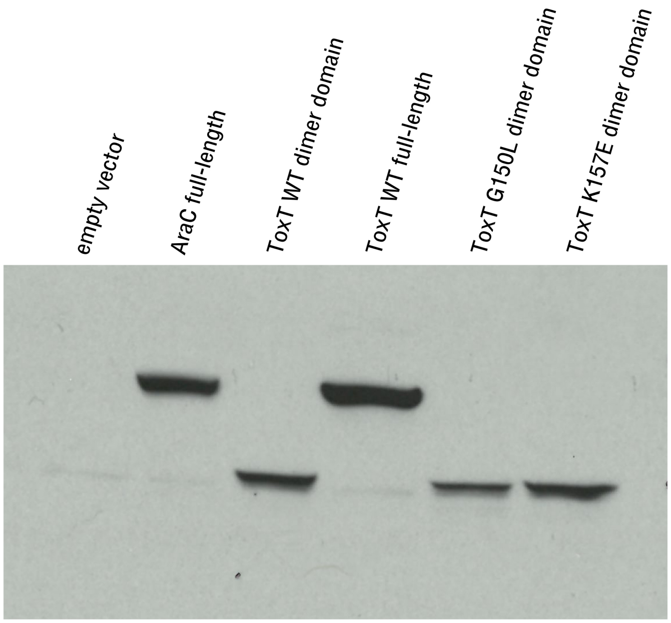


**Supplementary Figure 4.** Expression of the $ToxT_{EPI}$ LexA-fusions was confirmed by western blot. LexA DNA-binding domain western blots were performed on the cell lysates of each of the *sulA-lacZ E. coli* cultures used in the β-galactosidase assay presented in Figure 4.

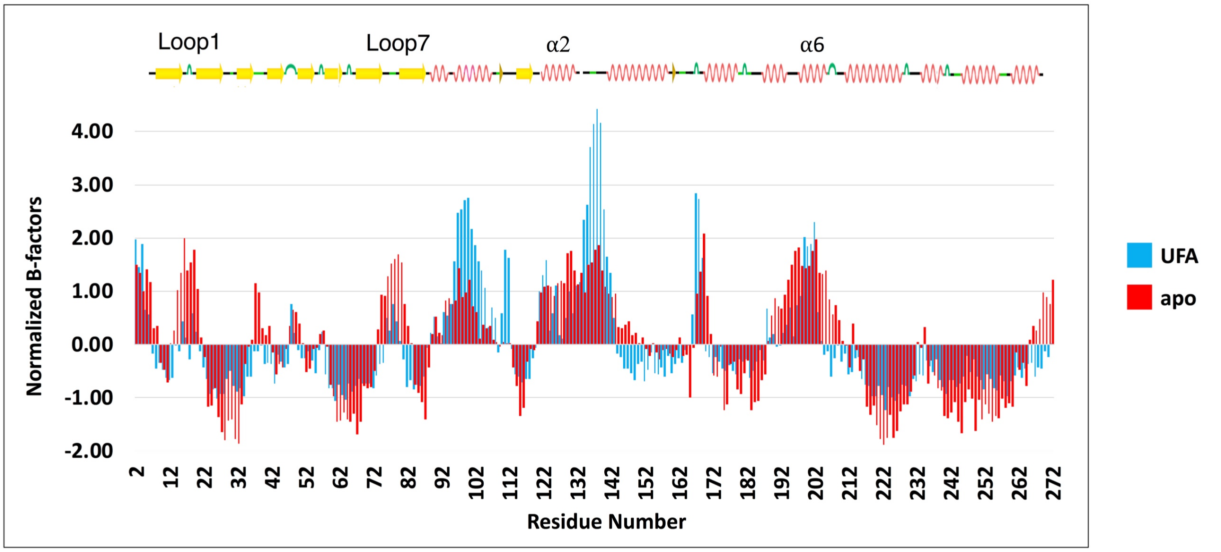


**Supplementary Figure 5.** Analysis of ToxT$_{ENV256}$ dynamics. Normallized crystallographic B-factors of UFA-bound (blue) and apo (red) ToxT$_{ENV256}$ structures. Normallized B-factors for each residue are calculated as the standard deviation from the mean B-factor of the structure. The secondary strucure of ToxT is shown above aligned to the corresponding residue number.

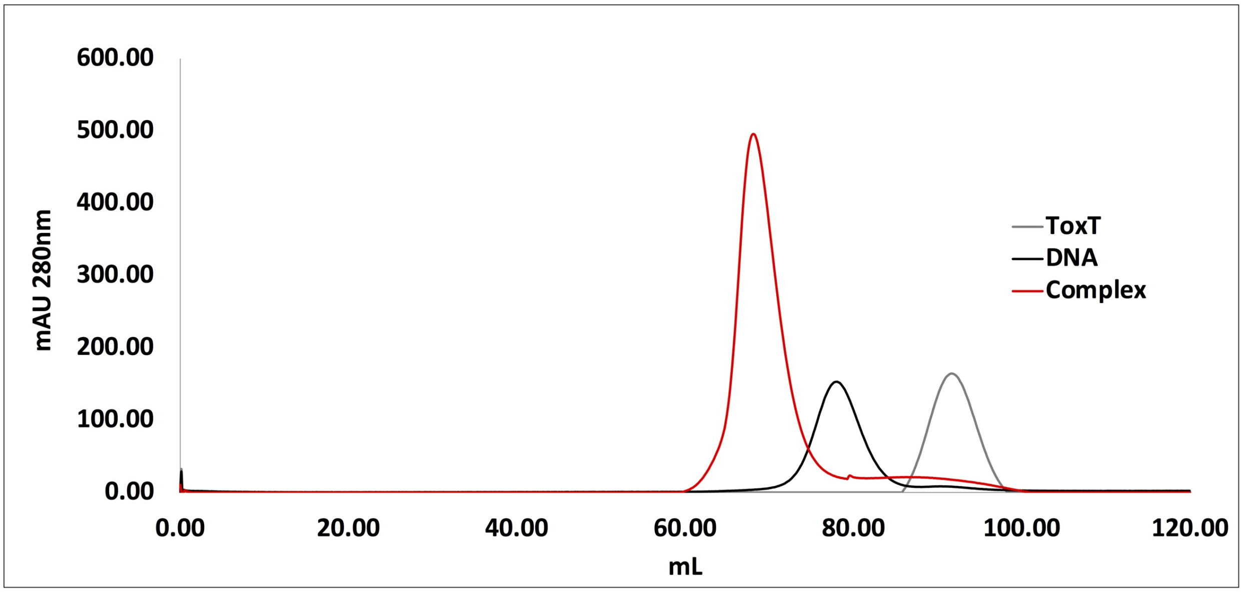


**Supplementary Figure 6.** Size Exclusion Chromatography of WT $ToxT_{ENV256}$-*ctx* complex.

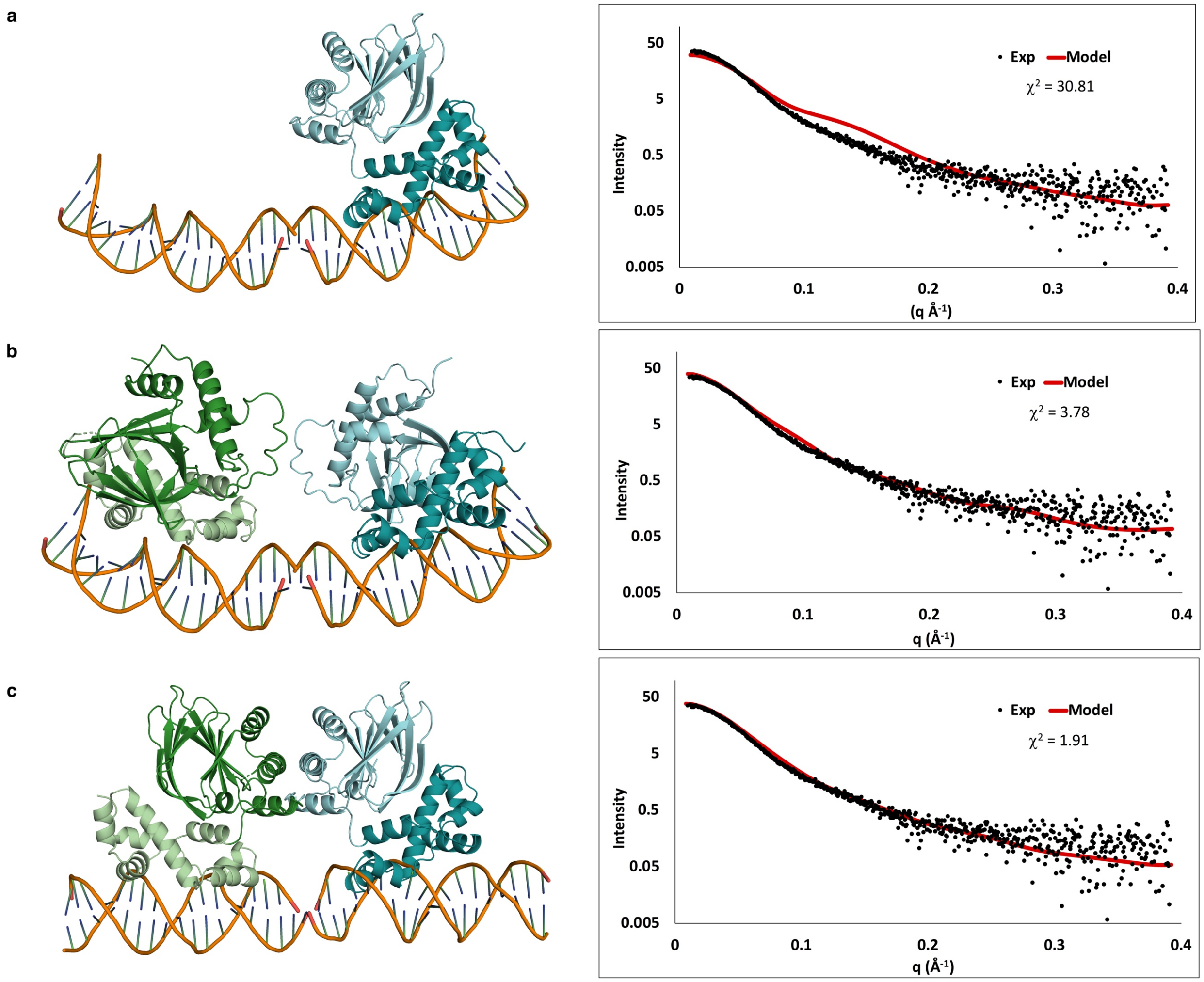


**Supplementary Figure 7.** Alternate models of the ToxT-DNA complex. Right, model, Left, calculated SAXS plot. **a**, Model of a single ToxT monomer bound to bent DNA ($\chi$=5.55). **b**, Model of two closed ToxT monomers bound to bent DNA ($\chi$=1.94). **c**, Model of an open ToxT dimer bound to straight DNA ($\chi$=1.38).

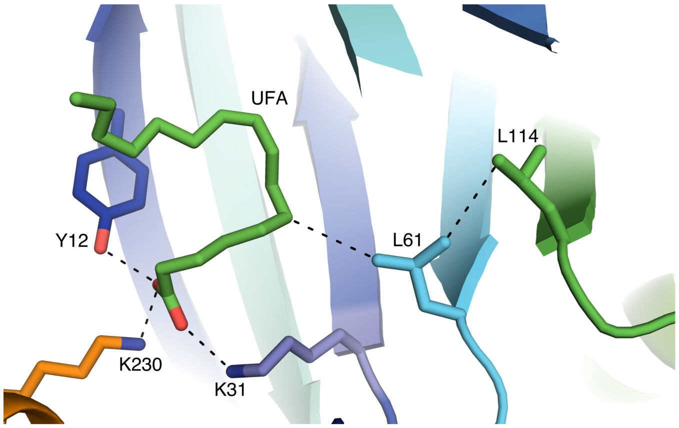


**Supplementary Figure 8.** Interactions between the unsaturated fatty acid and leucines 61 and 114 in $ToxT_{EPI}$.
