# Supplementary material for: Structural basis for virulence regulation in Vibrio cholerae by unsaturated fatty acid components of bile
Source: Commun Biol. 2019 Nov 28;2:440. doi: 10.1038/s42003-019-0686-x (PMC6882843; doi:10.1038/s42003-019-0686-x)
Supplement: Supplementary file 2 — Reporting Summary [file 42003_2019_686_MOESM2_ESM.pdf]

natureresearch

Corresponding author(s): Kull, F. Jon

Last updated by author(s): Oct 28, 2019

Reporting Summary

Nature Research wishes to improve the reproducibility of the work that we publish. This form provides structure for consistency and transparency in reporting. For further information on Nature Research policies, see Authors & Referees and the Editorial Policy Checklist.

Statistics

For all statistical analyses, confirm that the following items are present in the figure legend, table legend, main text, or Methods section.

n/a	Confirmed	
☐	☒	The exact sample size (*n*) for each experimental group/condition, given as a discrete number and unit of measurement
☒	☐	A statement on whether measurements were taken from distinct samples or whether the same sample was measured repeatedly
☒	☐	The statistical test(s) used AND whether they are one- or two-sided<br>*Only common tests should be described solely by name; describe more complex techniques in the Methods section.*
☒	☐	A description of all covariates tested
☒	☐	A description of any assumptions or corrections, such as tests of normality and adjustment for multiple comparisons
☐	☒	A full description of the statistical parameters including central tendency (e.g. means) or other basic estimates (e.g. regression coefficient) AND variation (e.g. standard deviation) or associated estimates of uncertainty (e.g. confidence intervals)
☒	☐	For null hypothesis testing, the test statistic (e.g. *F*, *t*, *r*) with confidence intervals, effect sizes, degrees of freedom and *P* value noted<br>*Give P values as exact values whenever suitable.*
☒	☐	For Bayesian analysis, information on the choice of priors and Markov chain Monte Carlo settings
☒	☐	For hierarchical and complex designs, identification of the appropriate level for tests and full reporting of outcomes
☒	☐	Estimates of effect sizes (e.g. Cohen's *d*, Pearson's *r*), indicating how they were calculated

Our web collection on statistics for biologists contains articles on many of the points above.

Software and code

Policy information about availability of computer code

Data collection	X-ray diffraction data was collected at the FMX beamline National Synchrotron Light Source II (NSLSII), Brookhaven National Laboratory, Upton, NY. A 1.8 Å data set of 1800 frames with an oscillation range of 0.2° was collected at a wavelength of 0.9790 Å with 0.1 second exposures at 100° K. The crystal to detector distance was 220 mm. The data set was indexed, integrated, scaled and merged using XDS. Data collection statistics are shown in Table 1.
Data analysis	The reflection file was converted and Rfree flags set (10% of unique reflections) using Phenix reflection file editor (35). The Matthew's coefficient was calculated, and it was determined that the asymmetric unit contained a single dimer of ToxTENV256. The structure of ToxTENV256 was solved by molecular replacement using Phenix Phaser-MR with ToxTEPI (3GBG) as the search model (36). Multiple rounds of refinement were carried out using Coot and Phenix.refine. Refinement statistics are shown in Table 1.

For manuscripts utilizing custom algorithms or software that are central to the research but not yet described in published literature, software must be made available to editors/reviewers. We strongly encourage code deposition in a community repository (e.g. GitHub). See the Nature Research guidelines for submitting code & software for further information.

Data

Policy information about availability of data

All manuscripts must include a data availability statement. This statement should provide the following information, where applicable:

- Accession codes, unique identifiers, or web links for publicly available datasets
- A list of figures that have associated raw data
- A description of any restrictions on data availability

Coordinates and structure factors have been submitted to the Protein Data Bank under accession numbers: 6P7R, 6P7T, and 6PB9 and this will be added to the revised manuscript. Table 1 contains all of the standard statistics associated with X-ray crystal structures. Error bars are indicated in Figure 4, which included n=3 experiments (n=3 will be added to the manuscript upon revision). A western blot of the LexAToxTEPI fusions confirming expression of protein presented in Figure 4 has been provided as Supplementary Figure 4.

Field-specific reporting

Please select the one below that is the best fit for your research. If you are not sure, read the appropriate sections before making your selection.

☒ Life sciences ☐ Behavioural & social sciences ☐ Ecological, evolutionary & environmental sciences

For a reference copy of the document with all sections, see nature.com/documents/nr-reporting-summary-flat.pdf

Life sciences study design

All studies must disclose on these points even when the disclosure is negative.

Sample size	na
Data exclusions	na
Replication	na
Randomization	na
Blinding	na

Reporting for specific materials, systems and methods

We require information from authors about some types of materials, experimental systems and methods used in many studies. Here, indicate whether each material, system or method listed is relevant to your study. If you are not sure if a list item applies to your research, read the appropriate section before selecting a response.

Materials & experimental systems

n/a	Involved in the study	
☒	☐	Antibodies
☒	☐	Eukaryotic cell lines
☒	☐	Palaeontology
☒	☐	Animals and other organisms
☒	☐	Human research participants
☒	☐	Clinical data

Methods

n/a	Involved in the study	
☒	☐	ChIP-seq
☒	☐	Flow cytometry
☒	☐	MRI-based neuroimaging
